# New York State Emergency Preparedness and Response to Influenza Pandemics 1918–2018

**DOI:** 10.3390/tropicalmed4040132

**Published:** 2019-10-30

**Authors:** Kay L. Escuyer, Meghan E. Fuschino, Kirsten St. George

**Affiliations:** Laboratory of Viral Diseases, Wadsworth Center, New York State Department of Health, Albany, NY 12208, USA; Kay.Escuyer@health.ny.gov (K.L.E.); Meghan.Fuschino@health.ny.gov (M.E.F.)

**Keywords:** emergency preparedness, influenza pandemic, surge support, just-in-time training, incident management system

## Abstract

Emergency health preparedness and response efforts are a necessity in order to safeguard the public against major events, such as influenza pandemics. Since posting warnings of the epidemic influenza in 1918, to the mass media communications available a century later, state, national and global public health agencies have developed sophisticated networks, tools, detection methods, and preparedness plans. These progressive measures guide health departments and clinical providers, track patient specimens and test reports, monitor the spread of disease, and evaluate the most threatening influenza strains by means of risk assessment, to be able to respond readily to a pandemic. Surge drills and staff training were key aspects for New York State preparedness and response to the 2009 influenza pandemic, and the re-evaluation of preparedness plans is recommended to ensure readiness to address the emergence and spread of a future novel virulent influenza strain.

## 1. The 1918 Pandemic Response

Leading the New York State (NYS) public health response to the 1918 “Spanish” influenza pandemic was the State Health Commissioner, Hermann M. Biggs M.D., who directed the State Department of Health to distribute posters, warning citizens of the severity of the epidemic with the following notification:

EPIDEMIC INFLUENZA

Help to protect yourself, your family and your town from a serious epidemic!

It is unlawful to cough or sneeze without turning the face away from others and covering the nose and mouth with a handkerchief, or to spit on the floor of any public place or public conveyance or on the sidewalk. A violation of this regulation is a misdemeanor and punishable by a fine or imprisonment or both. Epidemic influenza is conveyed solely from the discharges from the nose and throat.

Help to enforce the law! Save yourself and save others!

Hermann M. Biggs, M.D.

*State Commissioner of Health*

Biggs advised, “First, early detection of the disease; Second, immediate isolation of the patient under the direction of a physician or nurse...; Third, …general adoption of those rules of personal conduct by which alone the community can protect itself against the nose and mouth discharges of possible carriers; Fourth, the avoidance of crowded places…; Fifth, …more active and sustained attention to the cleansing of hands and belongings which may be contaminated” [1]. The New York State Public Health Council amended the Sanitary Code and declared epidemic influenza a reportable disease, effective 14 October 1918. Efforts were made to temporarily close crowded assemblies including schools, cinemas and theatres. These social and physical restrictions constituted the only preventative measures, as the bacillus influenza vaccine was ineffective, except to diminish some secondary bacterial infections. Medicines, including over the counter drugs, for symptom relief were also unavailable, and rest was considered the best treatment. Vital statistics data, military records and newspaper articles documented the devastation of the disease. The influenza epidemic subsided after an estimated 50 million deaths worldwide [2], including 650,000 deaths in the USA, and approximately 26,500 deaths in NYS [3]. Though the germ was unidentified at the time, retrospective analyses identified the causative agent to be influenza A/H1N1 [4].

## 2. Early Preparedness Efforts: Diagnostics, Surveillance and Vaccines 

**Figure 1:**
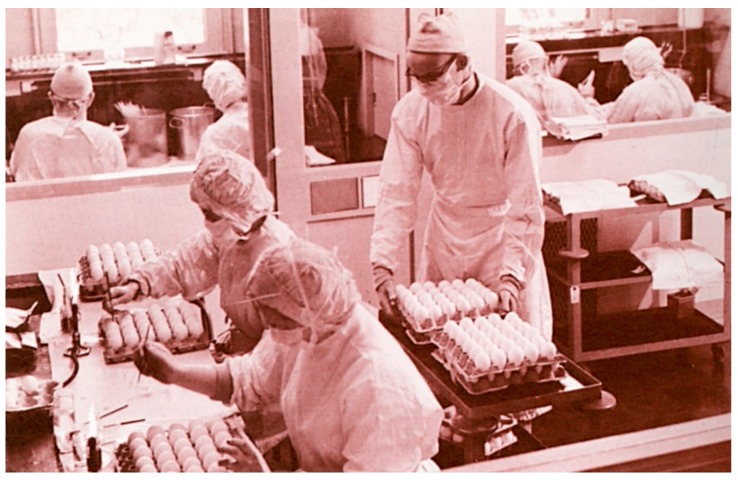
Staff at the New York State Department of Health, Public Health Laboratory (PHL), Division of Laboratories and Research, preparing eggs for inoculation with influenza A virus, to produce vaccines during the 1957–1958 influenza pandemic [9].

Essential to pandemic preparedness are components of influenza surveillance that detect the emergence and spread of influenza virus strains, as well as monitor morbidity and mortality. The efforts to surveil and control the spread of influenza were hampered until the discovery of the virus. First isolated in 1933 [5], the development of laboratory procedures for the culture and identification of influenza from clinical specimens also facilitated major research applications. Beginning in 1945, the New York State Department of Health (NYSDOH), Division of Laboratories and Research, now known as the Wadsworth Center, initiated laboratory research and diagnosis of viral disease. The construction of a new laboratory building for virology in 1949 bolstered the expansion of services for infectious viral agents. The laboratory responded to influenza outbreaks by isolating the virus and analyzing its antigenic capabilities for vaccination [6] (Figure 1). The test methods developed for respiratory specimens included complement fixation, hemagglutination-inhibition assays [6,7], and immunofluorescence assays for the identification of influenza viruses propagated in tissue culture [8]. 

Influenza vaccines were first licensed for commercial production in 1945 [10]. The propagation in embryonated chicken eggs [11] is still the most prevalent method for production. However, recent developments include an FDA-approved quadrivalent cell-based vaccine grown in the Madin Darby Canine Kidney (MDCK) cell line [12] and a trivalent recombinant influenza vaccine produced from a baculovirus expression vector in the Sf9 armyworm cell line [13]. These cell-based systems can be quickly scaled up for mass-production and circumvent the potential limited supply of eggs during pandemics when poultry availability may also suffer.

Concomitant with early technological advances was the establishment of national and international public health agencies: the World Health Organization in 1948, the Global Influenza Surveillance Network in 1952, and the US Communicable Disease Center (CDC) as a WHO Collaborating Center in 1956 [14]. For surveillance in NYS during the 1957 “Asian” influenza A/H2N2 pandemic and 1968 Hong Kong influenza A/H3N2 pandemic [4], influenza activity was tracked by school and workplace absenteeism and reported to the health department [15]. As the networks for surveillance and investigation of influenza were then global [16], and since the WHO tool for influenza virologic surveillance FluNet began in 1997, the surveillance data for influenza types, subtypes and lineages is updated weekly from 138 National Influenza Centers worldwide [17]. 

The events of 11 September 2001 prompted renewed national efforts for emergency preparedness and response. In addition to funds for biodefense, the U.S. Department of Health and Human Services issued $175 million to assist in preparedness for an influenza pandemic [18]. There was elevated concern for this due to the high case fatality rate in avian to human transmitted influenza A/H5N1 cases and the global spread of highly pathogenic avian H5N1 influenza virus. In 2006, as part the national Pandemic Influenza Plan [19], NYS released its Pandemic Influenza Plan [20] to guide public health officials and health care providers in preparedness and response to a potential influenza pandemic. One of the thirteen sections in this plan addressed the need for rapid and accurate laboratory testing of emerging influenza virus strains that may cause a pandemic. 

## 3. The 2007 New York State Influenza Laboratory Surge Drill

To evaluate laboratory preparedness for an influenza pandemic response, the Wadsworth Center designed and executed a surge drill from 26–28 June 2007, to test the public health laboratory (PHL) capacity in NYS. All four NYS PHLs participated: New York City, Westchester County, Erie County, and the New York State laboratory (Wadsworth Center) in Albany. 

For this surge drill, the participating PHLs estimated their maximum daily laboratory capacity for influenza testing. Mock influenza specimens, of which approximately 30% were positive, were prepared and shipped to the four laboratories, so that each received 25% of its estimated maximum capacity on each of three consecutive days. All specimens were sent with completed imitation request forms to facilitate test accessioning including the entry of patient demographics, followed by diagnostic testing and the reporting of results, for a thorough evaluation of the response capacity. A mock Incident Management System guided the participants to report daily on the results, facility logistics, scientific and technical issues, and resource management.

Facility logistics, laboratory testing, and waste stream management worked well at all sites. Personal protective equipment, assay kits, and reagent supplies were sufficiently available. The turn-around time from specimen receipt to result release was consistent with the normal workflow and result accuracy at the four laboratories ranged from 99.2% to 100%. 

The issues identified included the need for additional space for unpacking and accessioning specimens, more trained staff for accessioning, and additional automated platforms to maintain the workflow. After the exercise, two of the four participating laboratories reduced their estimated surge capacity. The drill raised additional questions such as how long laboratories could maintain working at maximum capacity; how they would manage other testing responsibilities; and whether staff availability would be impacted by their own illness, that of family members, or by school closures. The issues identified, lessons learned and how they were addressed were very useful during the pandemic that emerged two years later. 

## 4. The 2009 Influenza Pandemic

### 4.1. First Wave

**Figure 2:**
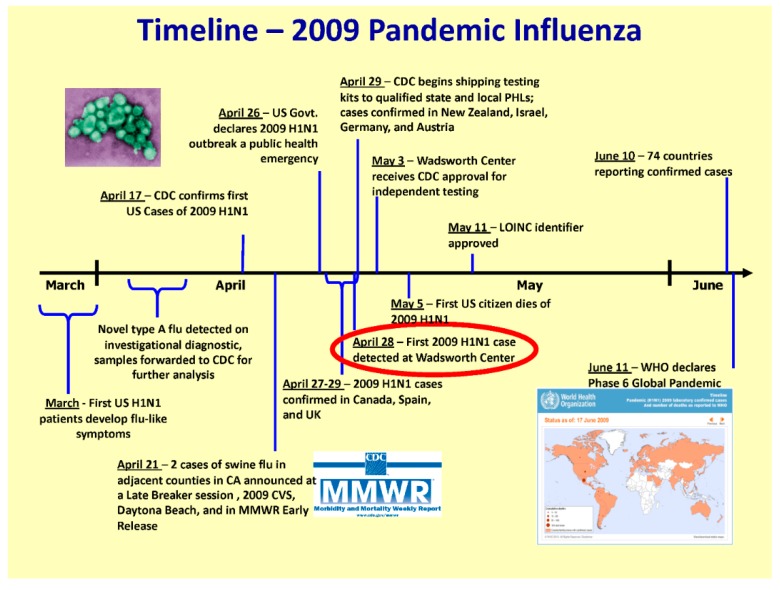
The timeline of the 2009 influenza pandemic and the emergency response by the international World Health Organization, the U.S. Centers for Disease Control and Prevention (CDC) and the NYS Wadsworth Center.

While state and national governments were primarily focused on the threat of an influenza pandemic with avian A/H5N1 from Asia, a novel A/H1N1 swine influenza strain emerged in Mexico with sustained human to human transmission in 2009 (Figure 2). 

The novel influenza A/H1N1 outbreak was declared a public health emergency in the USA on 26 April 2009. The NYSDOH implemented its Incident Management System to coordinate the response across all department subject matter leads including but not limited to: epidemiology, laboratories, immunization, planning, logistics, public affairs group, healthcare system management and division of administration. The NYSDOH was also active on the Interagency Taskforce on Influenza Preparedness during the 2009 pandemic.

**Figure 3:**
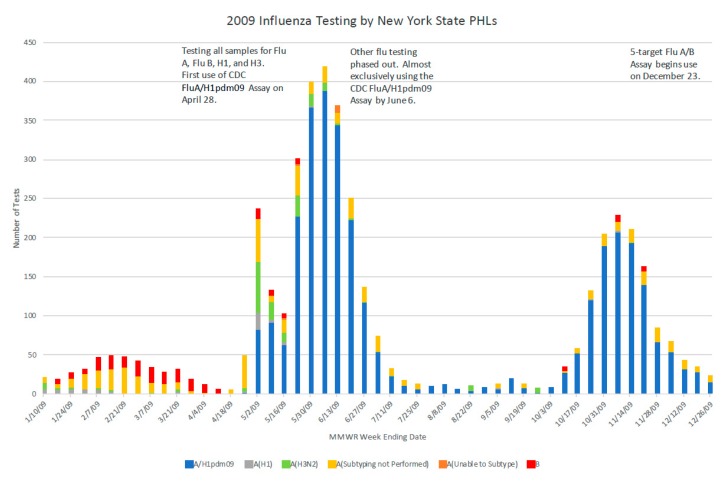
Testing by the Wadsworth Center in 2009 for influenza types and subtypes, identifying the pandemic influenza A/H1N1pdm09 strain once the specific CDC assay became available.

During the years of intense pandemic preparedness in the USA, the CDC developed a panel of human influenza real-time RT-PCR assays to detect influenza types A and B and distinguish subtypes A/H1N1 (seasonal) and A/H3N2. This panel was distributed to PHLs nationwide to ensure an all-state molecular detection capability. The A/H1N1pdm09 specific assay was added in 2009 to distinguish this virus from the previously circulating seasonal A/H1N1 influenza virus [21,22]. To assure reagent consistency and maintain supply, the reagents were later distributed to PHLs by CDC through a contracted agency. In NYS, as suspected case numbers rapidly surged in April 2009, state and local epidemiologists screened test requests, restricting testing to cases with true symptoms of influenza-like-illness, particularly to severe cases. This triage of cases was key to preventing the inappropriate application of the test. It also ensured that the laboratory workload did not become overwhelming. At the onset of the 2009 pandemic response at the Wadsworth Center, more space and automated equipment were allocated to accommodate the increased specimen load. The previous implementation of barcoding and electronic data transfer assisted in managing the testing surge (Figure 3). The Wadsworth Center also provided validation materials to clinical laboratories that were implementing laboratory-developed tests (LDTs), reviewed subsequent validation data for those A/H1N1pdm09 specific LDTs, and prepared proficiency testing panels. 

### 4.2. State and Federal Reporting

In 1918, epidemic influenza was made a reportable disease in New York State. On 1 December 2004, influenza was added to the NYSDOH reportable diseases list, requiring all NYS licensed clinical laboratories to report positive influenza test results to the NYSDOH via the Electronic Clinical Laboratory Reporting System. The results were reported to the CDC for inclusion in national surveillance, initially by online submission, and later by automatic electronic standardized messaging. During the 2009 pandemic, additional advisories directed that all “swine-origin influenza virus” [subsequently novel influenza A (H1N1)] test results from licensed clinical laboratories, whether positive, negative or indeterminate, be reported to state epidemiologists. This additional notification requirement did not continue after the pandemic. 

### 4.3. Surge Staff Training

The surge staff, previously trained in real-time PCR methods in other diagnostic laboratories at the Wadsworth Center, were reassigned to support and complement the work of Virology staff. In earlier years, a group of cross-trained staff had been maintained with knowledge and familiarity in virology as part of preparedness planning. However, over time, a different model was found to be more efficient. Rather than a cross-training mode of preparedness, where time and resources are continually tapped to train and maintain technical competencies for years, the Wadsworth Center opted for a just-in-time training mode, whereby the surge staff were trained as needed, for a limited set of skills necessary for performance of a defined task. The optimization of just-in-time training aligned the existing skills of staff with a related objective. The individuals and small teams each contributed their component in a production line to complete the entire testing process from sample receipt to test report. Overall, the just-in-time training process decreased costs while increasing work productivity.

### 4.4. Second Wave

By the second wave of the 2009 influenza pandemic in the Northern Hemisphere, clinical and commercial laboratories had introduced specific testing for the pandemic strain [23] and thus increased the national laboratory testing capacity. The priorities of the Wadsworth Center shifted to surveillance testing of sentinels, more severe hospitalized cases, and cases of suspected antiviral resistance.

With more contemporary communication methods than the distribution of posters used during the 1918 influenza pandemic, in 2009, mass media campaigns through television, radio, town hall meetings, social media, and the distribution of pamphlets, educated the public on the prevention of influenza transmission through good hygiene practices and vaccination. This was especially important in patients at high risk for severe disease, such as pregnant women. However, the novel influenza A 2009 pandemic quickly spread throughout America and the world. In NYS including New York City, the NYSDOH Bureau of Communicable Disease Control database showed a total of 53,930 laboratory confirmed influenza cases for 2009 [24]. These included 45,166 influenza A cases from 1 April to 31 December, the majority of which were influenza A/H1pdm09. The total influenza-associated fatalities in the USA during the first 12 months following the emergence of influenza A/H1N1pdm09 has been estimated at 12,469 [25], approximately 2% of the estimated fatalities experienced during the 1918 pandemic.

## 5. Lessons Learned for Pandemic Influenza Preparedness

On 22–23 June 2010, the Wadsworth Center hosted a statewide conference for clinical and public health laboratories to review the New York State response to the 2009 influenza pandemic. Central in the coordination of efforts and communication was the NYSDOH Incident Management System, which had been activated for 286 days, with daily state-wide calls to all services and relevant agencies. These statewide conference calls facilitated the sharing of experiences with state, regional and local health departments, identified limitations and problems, discussed the best laboratory practices, and regulatory issues that arose during the pandemic. Additionally, weekly conference calls had been held with state PHLs, the CDC and the Association of the Public Health Laboratories (APHL). 

A key tool for communication by the NYSDOH is the Health Commerce System, a secure web-based system for information exchange with state health agencies and health care facilities, providers and practitioners. The notifications, alerts and documents related to the novel A/H1N1pdm09 virus were sent electronically to enrolled health providers. Outreach from the NYSDOH Bureau of Communicable Disease Control to clinicians gave weekly updates of statewide influenza surveillance; directives for electronic reporting of positive cases; guidelines for suspected influenza-associated deaths; and guidelines for diagnosis of influenza. The discussions addressed the advantages and disadvantages of the rapid influenza diagnostic tests (RIDT), the culture of influenza viruses as well as molecular laboratory developed tests, and commercially available RT-PCR tests. The choice of antiviral medication was often based on surveillance data of RIDT results that indicated the circulating strain(s). The submission of specimens for laboratory testing was recommended if the results would impact clinical management. Antiviral resistance testing at the Wadsworth Center was advised for cases not responding to treatment, and antiviral medications were recommended for prophylaxis in certain situations. 

Previously established health care emergency preparedness plans detailing systems and actions contributed to the success of managing the pandemic. The continuity of operations planning (COOP) tools, for specifying alternative actions to maintain operations if the systems fail during a crisis, were useful to address the issues of staffing, facility support and integrity. A significant challenge during the pandemic was the concurrent continued global financial recession, which impacted resources and staffing in all health sectors. Good relations between state and local health departments, epidemiologists and infectious disease staff in hospitals and community settings were necessary to maintain calm. Ancillary activities, such as managing the enormous increase in phone calls, emails, notifications and paperwork, were taxing and required the designation of office staff to this work. Outreach and communication with submitters enhanced the specimen quality and corresponding patient information received in laboratories. Good statewide surveillance was critically dependent on physician participation. Prophylaxis with vaccines and medications had limited success in protecting the public and minimizing the spread of the pandemic. Despite the allocation of $1 billion by the U.S. Department of Health and Human Services in May 2009 [10], the availability of vaccines targeting the novel influenza A/H1N1pdm09 virus was too delayed to be an effective control. The distribution of available vaccines was prioritized by the department’s logistics section, leveraging the existing Strategic National Stockpile and Comprehensive Emergency Management Plans. The vaccine deployment activities included working with pharmacies and other private sector partners [20,26,27].

## 6. Present-Day Preparedness

The 2009 influenza pandemic demonstrated how preparedness plans and efforts are key in enhancing the public health agencies and health care providers ability to respond to an emergency. Good preparedness and response plans are designed to anticipate the needs and mitigate the consequences of major public health events, including a pandemic. Influenza pandemic plans need be comprehensive, workable, updated regularly, and integrated with a multi-hazard public health emergency plan for rapid implementation. The important components include command and control; surveillance and laboratory testing; healthcare planning; infection control; clinical guidelines; vaccine procurement, distribution, and use; antiviral medication procurement, distribution, and use; community mitigation; communications, training and education; workforce support; emerging influenza strains; and public health preparedness informatics [20]. The implementation of these planning components is considered at the local, regional and state level to help improve the response in a pandemic. The stockpiles for vaccines, personal protective equipment, supplies for diagnosis, and antiviral medications, need to be procured, maintained and packaged for distribution. Due to probable shortages of emergency responders and administrators, health care providers and laboratorians, the workforce may need to be supplemented with surge staff. Cross-training and just-in-time training alternatives can be considered for the various roles. Communication through information technology networks must be current, to enable prompt dissemination of risk assessments, guidelines, updates, and surveillance information. Information sharing between local responders and providers and local and state public health officials is imperative. Dialog can be facilitated between emergency management administration, public health agencies, and health care workers through emails, conference calls, and meetings; and for the community, through television, social media, and cell phone applications to send public service announcements and educational messages. Pandemic preparedness and response efforts need to be flexible to adjust to the waves of pandemic influenza, post-pandemic and interpandemic levels of influenza activity, and to the legal, ethical and policy issues within the jurisdictions [28].

The national and international networks for influenza surveillance for early detection continue to evolve. The World Health Organization has established six collaborating centers for epidemiologic and virologic surveillance [14], including the U.S. CDC. Since 2015, the Wadsworth Center Virology Laboratory houses one of three National Influenza Reference Centers for the CDC [29]. Additional National Influenza Reference Centers are in California and Wisconsin, and across the three centers which provide extensive analysis on approximately 3,000 positive influenza samples per season collected from all U.S. states and territories. For all specimens submitted to these reference centers, influenza viruses are sequenced directly with next generation sequencing, enabling the rapid identification of sequence drifts, shifts, new clades, resistance mutations and reassortments. This detailed analysis is essential for heightened rapid surveillance, vaccine selection and preparedness for seasonal influenza, as well as to detect the emergence of a novel influenza strain. Influenza viruses are also cultured and analyzed with classical assays.

Public health agencies must address the ongoing threats of potential influenza pandemics, such as the persistence of the highly pathogenic avian influenza virus A/H5N1 [30,31], and emergence of influenza A/H7N9 in 2013 [32,33]. Five H7N9 epidemics have occurred in China, the most severe arising in the 2016-2017 influenza season [34], although an extensive vaccination program appears to have controlled the situation at this time [35]. The influenza risk assessment tool (IRAT) has rated the Asian H7N9 virus as having the greatest potential pandemic risk amongst the novel influenza A viruses [36]. The indicators warn that the world is not ready for a pandemic [37], and surge plans need to be updated worldwide. Emergency preparedness and response efforts are critical in influencing the intensity and duration of the next influenza pandemic and its impact on public safety. In addition to the extensive use of rapid genomic surveillance tools for monitoring circulating viruses in the human population, it has become clear that a one health approach, with additional surveillance efforts in key animal species, is essential [38].
